# The signature of HBV-related liver disease in peripheral blood mononuclear cell DNA methylation

**DOI:** 10.1186/s13148-020-00847-z

**Published:** 2020-06-08

**Authors:** Kang Li, Ling Qin, Sanjie Jiang, Ang Li, Chi Zhang, Guihai Liu, Jianping Sun, Huanqing Sun, Yan Zhao, Ning Li, Yonghong Zhang

**Affiliations:** 1 Biomedical Information Center, Beijing You’An Hospital, Capital Medical University, Beijing, China; 2 Schools of Basic Medical Science, Capital Medical University, Beijing, China; 3 St. Edmunds College, Cambridge, CB3 0BN UK; 4 University of Oxford, Oxford, UK; 5 Clinical Laboratory Center, Beijing You’An hospital, Capital Medical University, Beijing, China; 6 Departments of Hepatobiliary Surgery, Beijing You’An Hospital, Capital Medical University, Beijing, China

**Keywords:** HBV-related liver disease, DNA methylation, LC predictive model, Fibrosis progression, Immune-related pathways

## Abstract

**Background:**

Hepatitis B virus (HBV)-related liver disease induces liver damage by hepatic immune and inflammatory response. The association between aberrant peripheral blood mononuclear cell (PBMC) DNA methylation and progression of liver disease and fibrosis remains unclear.

**Results:**

Here we applied Infinium 450 K BeadChip investigating PBMC genome-wide methylation profiling of 48 HBV-related liver disease patients including 24 chronic hepatitis B (CHB), 14 compensated liver cirrhosis (LC), and 10 decompensated liver cirrhosis (DLC). In total, there were 7888 differentially methylated CpG sites (36.06% hypermethylation, 63.94% hypomethylation) correlate with liver disease progression. LC was difficult to be diagnosed, intermediating between CHB and DLC. We used least absolute shrinkage and selection operator (LASSO)-logistic regression method to perform a LC predictive model. The predicted probability (*P*) of having LC was estimated by the combined model: *P* = 1/(1 − e^−x^), where *X* = 11.52 − 2.82 × (if AST within the normal range − 0.19 × (percent methylation of cg05650055) − 0.21 × (percent methylation of cg17149911 ). Pyrosequencing validation and confusion matrix analysis was used for internal testing, area under receiver operating characteristic curve (AUROC) of model was 0.917 (95% CI, 0.80–0.977). On the fibrosis progress, there were 1705 genes in LC compared with CHB, whose differentially methylated CpG sites loading within the “promoter” regions (including TSS1500, TSS200, 5′UTR, and the 1st exon of genes) subject into the enrichment analysis using Ingenuity Pathway Analysis (IPA). There were 113 enriched immune-related pathways indicated that HBV-related liver fibrosis progression caused epigenetic reprogramming of the immune and inflammatory response.

**Conclusions:**

These data support idea that development of HBV-related chronic liver disease is linked with robust and broad alteration of methylation in peripheral immune system. CpG methylation sites serve as relevant biomarker candidates to monitor and diagnose LC, providing new insight into the immune mechanisms understanding the progression of HBV-related liver fibrosis and cirrhosis.

## Introduction

Hepatitis B virus (HBV) infection is a common and growing global public health problem and CHB-related liver cirrhosis and hepatocellular carcinoma (HCC) are the cause of a high rate of morbidity and mortality [1]. WHO estimated that 257 million carriers of hepatitis B surface antigen (HBsAg) around the world in 2015, among which there were more than 887,000 deaths, mostly from hepatitis B resulted liver cirrhosis and HCC (World Health Organization fact sheets are available at www.who.int)

Clinically, it might be easy to distinguish decompensated liver cirrhosis (DLC) compared compensated liver cirrhosis (LC) according to presence of series of clinical symptoms form ascites, variceal bleeding, gastrointestinal bleeding, or hepatic encephalopathy, and Child-Turcotte-Pugh (CTP) score is class B or C [2]. It is relatively simple to diagnose DLC, although the treatment might be problematic. On the contrary, it is more challenging to make a distinction from CHB [3]. CT, MRI, and other imaging examinations are limited in the diagnosis of LC. It is still dependent on liver biopsy that is traditional gold standard procedure for staging of fibrosis and diagnosis of LC in Chinese guidelines for the prevention and treatment of chronic hepatitis B (version 2019) [4] and AASLD 2018 Hepatitis B Guidance [5]. However, it is invasiveness with risk of serious complications [6], sampling limitation, and interpretational variability [7]; therefore, clinicians have been trying to seek more accurate and noninvasive tools for assessing LC.

DNA methylation is stable epigenetic modification, whose variation is induced by environmental factors [8], and changes of DNA methylation are used to detect and monitor HBV-related chronic disease and HCC has been reported in recent years [9, 10]. Our previous studies had demonstrated that large changes in peripheral blood mononuclear cell (PBMC) DNA methylation altered in HBV/HCV-chronic hepatitis that might be playing a role in the progression form liver disease to HCC. Nevertheless, there was a dramatic and clear differentiation in PBMC DNA methylation profiles between chronic hepatitis and HCC. Meanwhile, the study supported the hypothesis that changes in DNA methylation of PBMC reflect changes in the host immune system interaction of HCC, rather than the footprints of circulating DNA of tumors or tumor substitutes [10].

Here, we initially applied Infinium Human Methylation 450 K BeadChip arrays to examine genome-wide DNA methylation profiles in PBMC samples from 48 HBV-related liver fibrosis and cirrhosis. The chip covered about 480,000 single CpG sites from 21,231 human genes annotated by the university of California Santa Cruz genome database [11]. Genome-wide CpG sites methylation profiling of PBMC DNA could provide relevant biomarker candidates to monitor the process of HBV-related liver fibrosis and cirrhosis, which was used to examine the associations of LC specific CpG sites for establishing diagnosis model. Meanwhile, methylated variations of CpG sites occurred mostly within the promoter regions of genes to regulate their transcription [12]. CpG sites methylation correlating with liver fibrosis and cirrhosis could contribute evidence for immune functional and canonical pathway changes. Our findings also provided new insight into the immune mechanisms underlying the progression of HBV-related liver fibrosis and cirrhosis.

## Result

### Clinical and pathological characteristics of the diseased patients

Forty-eight patients with HBV-related diseases were from Beijing area, whose clinical and pathological characteristics were described in Table 1. These three groups did not differ for age, gender, smoking and alcohol, total bilirubin (TBIL), aspartate transaminase (AST), alanine transaminase (ALT), and serological diagnosis model (APRI, FIB-4) (all *P* > 0.05). All patients were subjected accurate diagnosis of staging of fibrosis and cirrhosis with traditional gold standard liver biopsy.

**Table 1:** Clinical and pathological characteristics of patients with HBV-related diseases

Variable	CHB (*n* = 24)	LC (*n* = 14)	DLC( *n* = 10)	*P* value
Age (mean ± SD)	42.5 ± 6.4	43.1 ± 7.2	41.3 ± 10.0	0.848
Gender				0.953
Male/female	16 (66.7%)/8	10 (71.4%)/4	7 (70.0%)/3	
Smoking				0.181
No	12 (50%)	5 (35.7%)	4 (40.0%)	
Infrequent	7	2	3	
Heavy	5	7	3	
Alcohol				
No	15 (62.5%)	9 (64.3%)	8 (80.0%)	0.181
Infrequent	7	1	2	
Heavy	2	4	0	
TBIL (μmol/L)	25.63 ± 25.87	41.75 ± 46.23	70.04 ± 105.96	0.133
AST (U/L)	127.76 ± 182.01	332.8 ± 576.45	76.2 ± 103.7	0.131
ALT ((U/L))	206.03 ± 319.22	524.14 ± 956.423	73.09 ± 117.38	0.130
APRI (mean ± SD)	77.75 ± 109.58	269.08 ± 475.83	91.53 ± 71.21	0.108
FIB4 (mean ± SD)	2.15 ± 1.42	3.63 ± 4.54	4.95 ± 3.31	0.054
HBsAg positive/negative	23 (100%)/0 (a)	12 (85.7%)/2	9 (100%)/0 (b)	0.083
Anti-HBs positive/negative	1 (4.16%)/22	2 (14.29%)/12	0 (0%)/9	0.291
HBeAg positive/negative	16 (69.56%)/7	6 (45.85%)/8	2 (22.22%)/7	0.034
Anti-HBe positive/negative	10 (43.47)/13	8 (57.14)/6	6 (66.67%)/3	0.447
Anti-HBc positive/negative	23 (100%)/0	14 (100%)/0	9 (100%)/0	NA
Fibrosis				
S1	10			
S2	9			
S3	5			
S3-4		4		
S4		10	10	

*CHB* chronic hepatitis B, *LC* compensated liver cirrhosis, *DLC* decompensated liver cirrhosis, *TBIL* total bilirubin, *AST* aspartate transaminase, *ALT* alanine transaminase, *APRI, FIB-4* serological diagnosis model, *HBsAg* hepatitis B surface antigen, *Anti-HBs* hepatitis B surface antibody, *HBeAg* hepatitis B e antigen, *Anti-HBe* hepatitis B e antibody, *Anti-HBc* hepatitis B core antibody. a: one CHB patient data of HBV markers unavailable, b: one DLC patient data of HBV markers unavailable

### Correlation between quantitative distribution of site specific DNA methylation levels and progression of liver disease

**Fig. 1:**
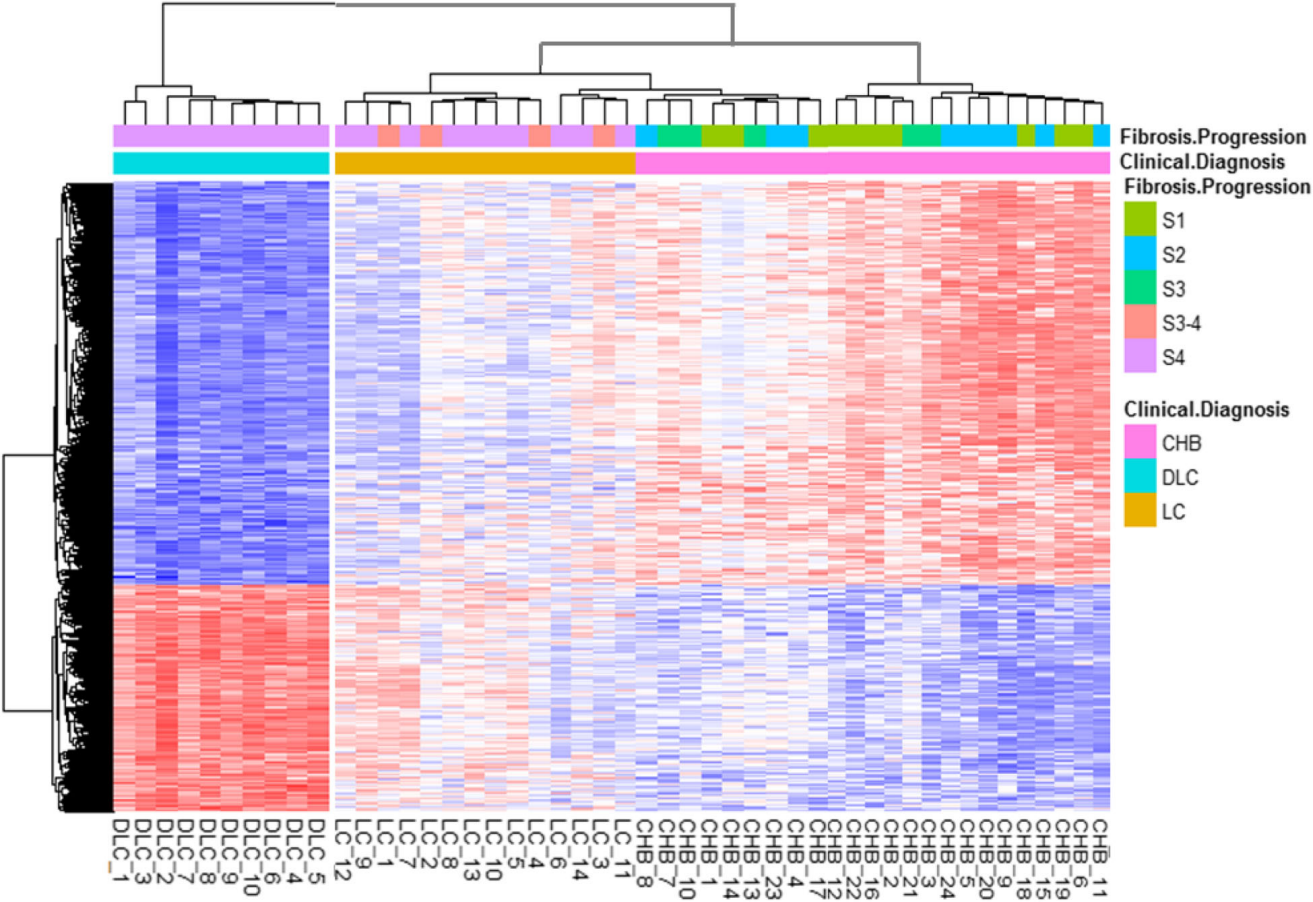
Heatmap showing hierarchical clustering based one minus Pearson correlation of 48 liver disease patients by beta values of 7888 CpG sites

PBMC was isolated by density gradient centrifugation from 48 patients’ blood. After treatment by bisulfite conversion, PBMC genomic DNA was detected using Illumina Infinium HumanMethylation450 BeadChip arrays. Raw data was loaded and analyzed using the ChAMP Bioconductor package in R. In total, 485,577 methylation probes were in the 450 K arrays, while probes with a detection *P* value > 0.01 (1850 probes), with a beadcount < 3 in at least 5% of samples (6368 probes), containing single nucleotide polymorphisms (SNPs, 49,659 probes), aligning to multiple locations (7074 probes), locating on X,Y chromosome (10,073 probes) as well as NoCpG sites (3330 probes) were consequently removed, finally 407,223 probes were used for further analysis. Following normalization and correction for batch effects, Pearson correlation analysis with Bonferroni correction for multiple testing (< 1 × 10^−7^) was used to show linear correlation between the quantitative distribution of CpG sites methylation levels across the array and liver disease progression. The result revealed significant correlation between CpG sites methylation levels and progression of liver disease. DNA methylation levels obviously varied in the whole genome, within 7888 CpG sites (*r* > 0.8, *r* < − 0.8; *P* < 10^−7^, Supplementary table 1) including 2845 (36.06%) hypermethylation CpG sites and 5043 hypomethylation CpG sites most significantly changed during liver disease progression. Hierarchical clustering analysis showed 7888 differentially methylated CpG sites probes distinguish patients of CHB, LC, and DLC (Fig. 1). Obviously, DLC patients were differed form CHB and LC ones, in addition to fibrotic process of the liver, the other symptoms of DLC might be major factors distinguishing DLC from of CHB and LC. Meanwhile, liver fibrosis S4 stages were both within DLC and LC patients who were separated into two groups, indicating that liver fibrosis was not the cause but one of outcome of the liver disease.

### The PBMC DNA methylation characters of progression of liver disease remained significant after correction for potential confounders: gender, age, smoking, alcohol drinking, and cell type.

To rule out the influence of cell mixture distribution in PBMC, gender, age, smoking, and alcohol drinking on DNA methylation, these covariates were checked for correction. First of all, the cell mixture distribution for each patient was determined using the Houseman algorithm [13]. There was no significant difference in cell mixture between the groups using two-way ANOVA followed by pair-wise comparisons and false discovery rate. Then, a multivariate linear regression was performed on the normalized beta values of the 7888 CG sites to divide all cases using group (CHB, LC**,** and DLC), gender, age, smoking, alcohol drinking, and cell type as covariates. In the model including the other covariates, all CG sites remained highly significant for group covariate, even after following Bonferroni corrections, all CG sites remained highly significant for the group (Supplementary table 2).

A multifactorial ANOVA analysis was performed on the beta values of the 7888 sites as dependent variables and group (CHB, LC**,** and DLC), gender, age, smoking, and alcohol drinking as independent variables to determine whether there were possible separate and combined effects on DNA methylation by the five independent variables. The group remained significant for all 7888 CG si**t**es, no significant interactions between group and separate or combine other four independent variable**s** (gender, age, smoking, and alcohol drinking) were found after Bonferroni corrections (Supplementary table 3).

### Profiles and cluster of CpG sites methylation level by STEM analysis

**Fig. 2:**
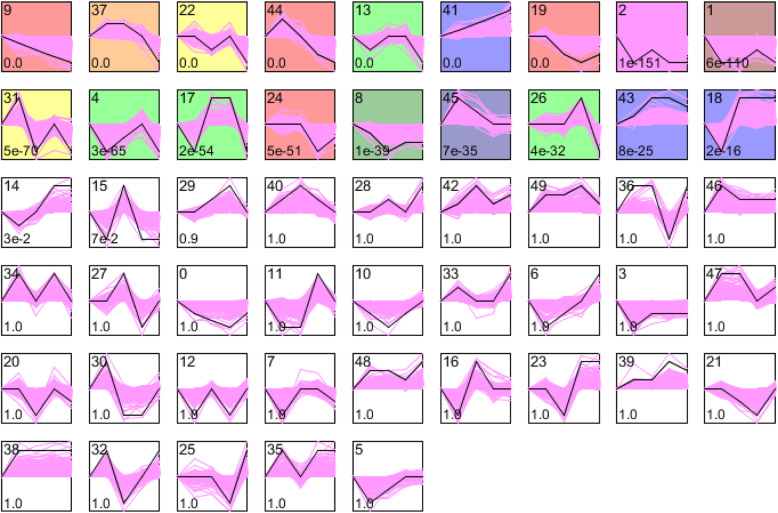
STEM clustering was used to analyze profiles and cluster of methylation level of CpG sites. The upper numbers indicate type of profiles. The lower contained *P* value of the profiles. The black lines showed mean pattern of CpG sites methylation level of the profile. The pink lines showed all CpG sites methylation level of profiles. Colorful background showed significantly different profiles. The *x*-axis represents 5 stages (S1, S2, S3, LC, DLC), and the *y*-axis represents CpG sites beta values

We aimed to address the hypothesis that changes of DNA methylation level reflected the progression of the disease. We used **s**hort **t**ime-series **e**xpression **m**iner (STEM) analysis to perform a global temporal analysis of the detected CpG sites, in order to find the sites demonstrating a common pattern of change of methylation level along with the disease stages. Of the 50 randomly selected profiles investigated, 18 profiles (1, 2, 4, 8, 9, 13, 17, 18, 19, 22, 24, 26, 31, 37, 37, 43, 44, and 45) showed a statistically significant higher number of CpG sites than expected (shown as colored profiles in Fig**.**2). Among these profiles, we focused on two profiles for further analysis. In profile 24, the methylation level of the CpG sites was stable from stage S1 to stage S3, then reached the lowest level at stage LC, afterwards returned to higher methylation level at stage DLC (but still lower than stage S3). In profile 26, the methylation level of the CpG sites was stable from stage S1 to stage S3, and then reached the highest methylation level at stage LC. At stage DLC, the methylation level was the lowest.

### Selection of from PBMC DNA CpG sites for LC prediction model

**Fig. 3:**
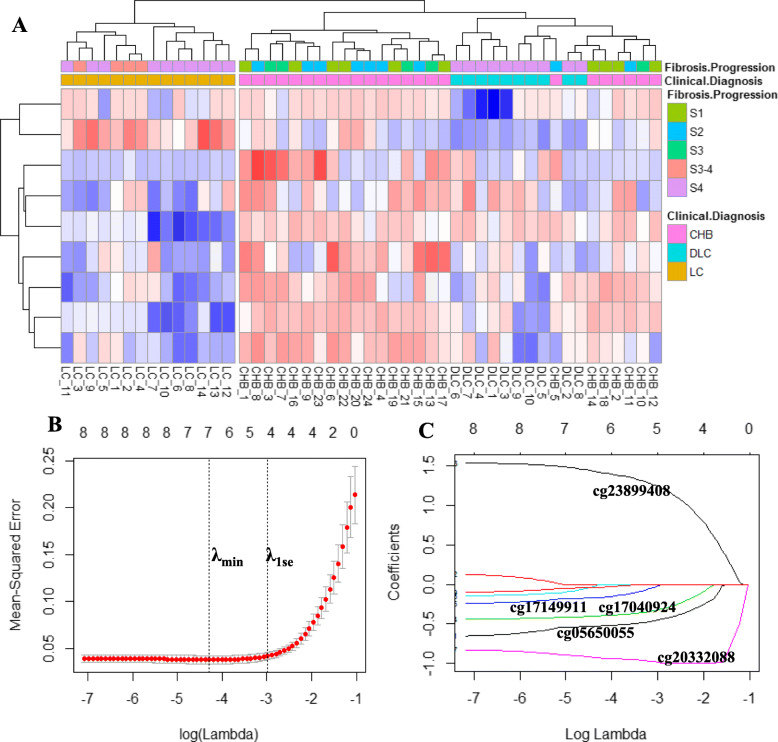
Biomarker CpG sites selection using hierarchical clustering and LASSO-regularized regression. **a** Hierarchical clustering analysis consisting of nine differentially CpG sites in PBMC DNA from 48 liver disease patients. **b** Representative repetition of five-fold CV LASSO coefficients of nine candidate CpG sites. The first vertical dotted line corresponds to the *λ*_min_ that minimized binomial deviance during CV. The second dotted line corresponds to *λ*_1se_ (0.051), used for the selection of biomarker CpG sites. **c** LASSO coefficient profile plot of the coefficient paths. Nine CpG sites had their coefficients significantly different from zero

From profile no. 24 and no. 26, nine CpG sites were selected and successfully distinguish LC PBMC samples from CHB and DLC samples using hierarchical clustering. Nine CpG sites candidates used to subject least absolute shrinkage and selection operator (LASSO) regularized regression method [14] implemented in the glmnet R package [15, 16] to identify CpG sites panel that predicted LC. Optimal value of LASSO penalty weighting, *λ* was selected with 5-fold cross-validation (CV). The minimum-deviance (*λ*_min_) plus 1 standard error (*λ*_1se_) was used to select CpG sites sets (Fig. 3a). Using the *λ*_1se_ = 0.051, five CpG sites cg23899408 (*HOOK2*), cg20332088 (no gene can be mapped), cg17040924 (*OR52M1*), cg17149911 (*AAK1*), and cg05650055 (*MYEOV*) were prepared for LC prediction model (Fig. 3c).

### LC prediction model from CpG sites in PBMC DNA combined with AST in blood

**Fig. 4:**
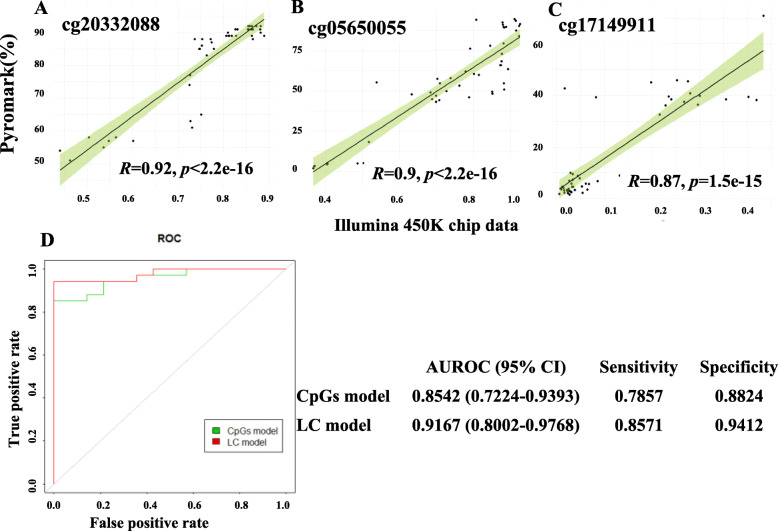
Correlations between Illumina 450 K Array data and PyroMark sequencing data of three CpG sites, **a** cg20332088, **b** cg05650055 (*MYEOV*), **c** cg17149911(*AAK1*), **d** ROC curves of the CpG sites (cg17149911 + cg05650055) model and LC-combined model, and their respective AUROC, 95% CIs, sensitivity and specificity were reported in parentheses.

Firstly, CpG sites methylation obtained from Illumina 450 K array data were validated using the correlation analysis. The three CpG sites (cg20332088, cg05650055, and cg17149911) were chosen for pyromarksequencing, and the primers and cycling conditions for pyrosequencing were shown in Supplementary table 4. The results showed remarkable correlation between quantitation of CpG sites methylation ratio using pyrosequencing and Illumina 450 K array data, with a correlation coefficient of 0.92, 0.90, and 0.87, respectively (Fig. 4a, b, and c).

Univariate analysis and multivariate logistic regression were performed on five CpG sites were selected by LASSO and clinical characteristics of the patients to determine the independent association of each variable with LC. In consideration of clinically relevant and given the number of events available [17], thus the final model contained three variables: cg05650055 (PyroMark value), cg17149911 (PyroMark value), and AST levels in blood. The predicted probability (*P*) of having LC was estimated by the combined model: *P* = 1/(1 − e^−x^), where *X* = 11.52 − 2.82 × (if AST within the normal range) − 0.19 × (percent methylation of cg05650055) − 0.21 × (percent methylation of cg17149911) (Table 2).

**Table 2:** Multivariate logistic regression model to predict LC

Variables		B	OR	95% CI of OR	*P*
Intercept		11.52	1.01 e + 05		0.0326
cg05650055 (%)		− 0.19	0.83	0.68–0.92	0.0386
cg17149911 (%)		− 0.21	0.81	0.64–0.92	0.0471
AST (U/L)	≤ 15 or > 40(male) ≤ 13 or > 35(female)	Reference	Reference		
	15–40 (male)	− 2.82	0.059	0.003–0.538	0.05
	13–35 (female)				

*B* regression coefficient, *OR* odds ratio in favor of having LC

The LC prediction model was conducted for training and internal testing with 48 liver disease patients and its accuracy was examined using a confusion matrix analysis. The AUROC of CpG sites (cg17149911 + cg05650055) model and LC model to predict LC was 0.8542 (95% CI, 0.722–0.939), 0.917 (95% CI, 0.80–0.977), respectively in Fig. 4d and Supplementary table 5.

### The PBMC DNA methylation characters between LC and CHB remained significant after correction for potential confounders: gender, age, smoking, alcohol drinking, and cell type.

**Fig. 5:**
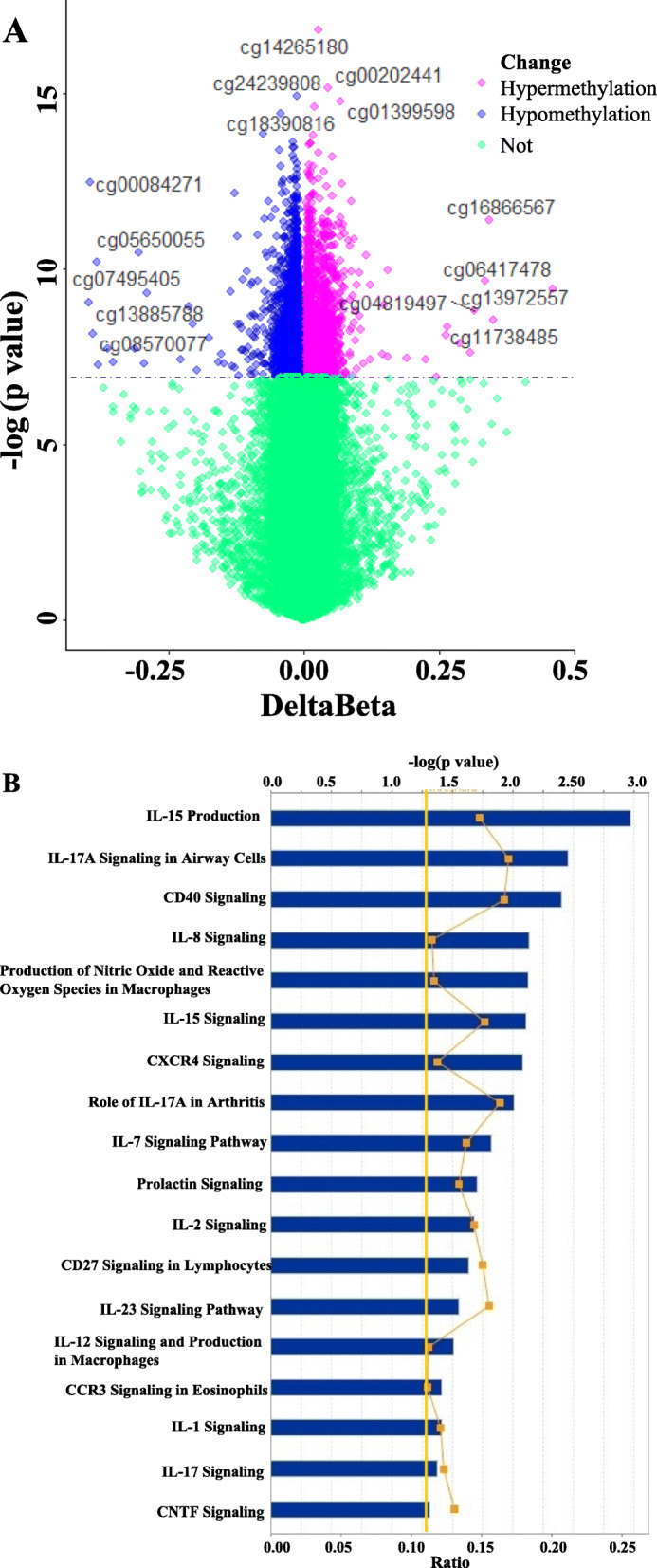
**a** The volcano plot for differential DNA methylation CpG sites between LC and CHB. The *x*-axis showed the mean DNA-methylation difference (delta Beta), whereas the *y*-axis showed the –log10 of the adjusted *P* value, hypermethylated CGs were shown in red, hypomethylated CGs were shown in red blue, non-significant methylated change CGs were shown in green (Bonferroni correction *P* value < 0.05). **b** Immune functional and canonical pathway changes between LC and CHB. Ten most significant pathways identified by the IPA canonical pathways analysis (“Cellular Immune Response,” “Cytokine Signaling,” and “Humoral Immune Response”) of genes, whose “promoter” regions containing differentially methylated CpG probes. Upper *x*-axis represented negative –log (*P* value) of the enrichment score, which calculated by IPA using Fisher’s exact test, right-tailed. Lower *x*-axis represented the ratio values between selected genes and the total number of genes in each of these curated pathways, and the orange curve pointed out the ratio, orange vertical line represented threshold value was 1.3.

The differentially methylated CpG sites between LC and CHB were identified by applying the Bioconductor package Limma. The result showed 4325 significantly methylated CpG sites (Bonferroni correction *P* value ≥ 0.05), including 1949 (45.06 %) hypermethylated and 2376 (54.94%) hypomethylated CpG sites (Fig. 5a) in PBMC DNA of LC**.**

A multivariate linear regression was performed on the normalized beta values of the 4325 CG sites to differentiate LC from CHB cases using group (CHB versus LC), gender, age, smoking, alcohol drinking, and cell type as covariates. In this model including the other covariates, all CG sites remained highly significant for group covariate, following Bonferroni corrections, 4219 CG sites remained highly significant for the group (Supplementary table 6).

A multifactorial ANOVA analysis was performed on the beta values of the 4325 sites as dependent variables and group (CHB versus LC), gender, age, smoking, and alcohol drinking as independent variables to determine whether there were possible separate and combined effects on DNA methylation by the five independent variables. Following Bonferroni corrections, 4167 CG sites remained highly significant for the group, 158 significant interactions between group and separate or combine other four independent variables (gender, age, smoking, and alcohol drinking) were found (Supplementary table 7).

### Immune functional and canonical pathway changed between LC and CHB in PBMC

To gain insight into the immune functional footprint of the differentially methylated genes between LC and CHB in PBMC, the genes whose “promoter” regions containing differentially methylated CpG probes (Supplementary table 8) were subjected to enrichment analysis using Ingenuity Pathway Analysis (IPA). There were 113 enriched immune-related pathways (Supplementary table 9), and the highlighted pathways (*P* value < 0.05) with relevance to HBV-related fibrosis progression were identified in this analysis (Fig. 5b). The top five immune functional and canonical pathways were including IL-15 production, IL-17A signaling in airway cells, CD40 signaling, IL-8 signaling, production of nitric oxide, and reactive oxygen species in macrophages.

## Discussion

HBV infection does not cause directly hepatocyte lesions, interactions between virus and host immune response determine virus clearance or liver damage [18]. It has been well known of viral clearance; however, mechanisms of host immune response responsible for the liver damage caused by HBV infection are still need to elucidate. Less is known about the alterations occurring in immune cells DNA methylation in non-tissues in HBV-related disease patients. From our analysis, 7888 CpG sites in PBMC DNA whose quantitative state of methylation showed strong correlation (*r* > 0.8, *r* < − 0.8; *P* < 10^−7^) with progression from CHB, LC to DLC (Fig. 1), which remained significant even after taking into account in the regression model differences in gender, age, smoking, alcohol drinking, and cell-type distribution. These data support idea that development of HBV-related chronic liver disease is linked with robust and broad alteration of methylation in peripheral immune system.

Interestingly, the overall of differences in PBMC DNA methylation varied as HBV-related chronic disease advanced, from hypermethylation to hypomethylation in Fig. 1. Importantly, the profiles of PBMC DNA of DLC patients were clearly differentiated from the CHB and LC ones. There was a sharp boundary between DLC and CHB and LC in heatmap, even both LC and DLC were fibrotic S4. Except for fibrosis level, the clinical and pathological characteristics of DLC might be other important factors distinguishing themselves from CHB and LC. This inference was consistent with some clinical observation that there was no significant correlation between severity of necrosis or liver injury and fibrosis progression in DLC patients [19, 20]. The same was true in IL-15RαKO mice [21].

The alteration of PBMC DNA methylation modification with chronic liver disease progression is important and helpful to find biomarkers of liver disease in clinical diagnosis and therapies [22]. Clinically, it might be easy to distinguish DLC according to a series of clinical symptoms. However, the LC patients with fibrosis S4 stage are difficult to make a distinction from CHB [3]. At present, LC diagnosis is still dependent on liver biopsy that is traditionally gold standard procedure for staging of fibrosis and diagnosis. LC specifically PBMC DNA-methylated CpG sites were significantly enriched by STEM analysis based on their beta value of differentiation in the process of liver fibrosis progression [23]. Based on the both profile no. 24 and no. 26 form STEM results, we used five-fold cross-validation to select penalty weighting *λ*_min_, five candidate CpG sites were selected by LASSO basing on *λ*_1se_. In consideration of clinically relevant and available number of events, we selected cg05650055 and cg17149911 to prepare for LC prediction model. Considering together with clinical characteristics of patients, we made the LC combine model. In this study, confusion matrix analysis was used for LC model internal testing with 48 liver disease patients, further external validation will carry out to confirm detection efficiency of model.

There were 4325 differentially methylated CpG sites in PBMC DNA of LC comparing CHB, including 1949 (45.06 %) hypermethylation and 2376 (54.94%) hypomethylation CpG sites. Previous reports demonstrated 18,234 significant CpG sites in biopsy DNA of severe fibrosis comparing mild; however, hypermethylated CpG sites (11,475, 62.94%) were more than hypomethylated ones (6759, 37.06%) [9]. The DNA methylation profile of PBMC DNA was not the same as tissue in severe fibrosis patient. Without an overlap differentially methylated CpG site between PBMC and liver biopsies (partial available data) suggested that changes of DNA methylation seen in PBMC reflect variation in the immune system were not a footprint of disease tissue.

The accurate relationship between DNA methylation and gene expression was complex and unclear [24, 25]. An effect of DNA methylation on transcription was highly dependent on specificity of tissue and genomic context [24]. Methylation of promoter regions (TSS1500 and TSS200) and 5′UTR had mostly negative regulatory effect on transcription compared to gene body regions [26, 27]. The 1st exon was considered as an important negative factor of transcription regulation [28]. Hypermethylation of the 1st exon region blocked transcriptional initiation, or vice versa and its length was closely related with gene activity [28–30]. On other hand, once transcription began, methylation of downstream region was not a significant interference factor restraining extension of RNA polymerase [28]. Methylation of CpGs loaded within the body regions, 3′UTR, ExonBnd, and intergenic region was not taken into account for functional and canonical pathway analysis. Finally, we selected CpGs loaded within “promoter” regions including TSS1500, TSS200, 5′UTR, and the 1st exon of genes for immune functional and canonical pathway analysis.

IPA canonical pathways analysis showed the immune and signal pathway had changed in LC compared to CHB, and provided an overview of the functional pathways that were affected. IL-15 and its signaling played an anti-fibrotic role in two independent ways, IL-15 maintained homeostasis of NK cell whose cytolytic effect avianized fibrogenic potential of hepatic stellate cells (HSCs) [31] and IL-15Rα inhibited collagen transcription repressors in HSCs [21]. In hepatitis B virus-related cirrhosis, IL-17 could activate STAT3 signaling pathway to induce production of type I collagen in HSCs [32]. IL-17A played critical role of HSC activation for liver fibrosis, and the induction of inflammatory cytokine and neutrophils recruitment were IL-17A-dependent [33]. Activation of the IL-17 pathway was a characteristic of human alcoholic hepatitis [34]. IL-8 significantly increased in chronic liver diseases, it served as a novel role to recruit and activate hepatic macrophages via CXCR1 that enhanced hepatic inflammation [35]. The production of nitric oxide (NO) and reactive oxygen species (ROS) in macrophages suggested that except for neutrophils [36], macrophages were another important source of NO and ROS in fibrosis progression. The CXCL12/CXCR4 pathway recruited and retained immune cells to involve into inflammation and fibrosis both in chronic HCV and HBV infection [37]. These findings provided considerable support for the point that the epigenetic altering of immune system and inflammatory response during HBV-related liver fibrosis progression.

We build a LC prediction combine model, using confusion matrix analysis to confirm the model to be highly sensitive and specific. An external verification study was a necessary for LC diagnosis. The progression of HBV-related liver disease is affected by interaction between viral load, viral genotypes, and immune system. We determine that the PBMC DNA methylation characters of progression of liver disease (7888 CGs) and difference between LC and CHB (4325 CGs) remain significant after correction for HBV markers (Supplementary table 10 and 11) and PBMC cell type (CD4+ T cell, CD8+ T cells, B Cells, natural killer cells, monocytes, granulocytes). However, further research needs to investigate the effect of HBV markers’ change, viral genotype, and HBV infection of PBMC on methylation variety in host PBMC. Meanwhile, we still need to find out the specific methylation site in PBMC of HBV infection compared with cases associated with HCV infection, especially end-liver diseases.

In conclusion, we demonstrated epigenome-wide methylation of fibrosis and cirrhosis in PBMC DNA of HBV-related liver disease patients. From PBMC DNA methylome profiling, we found that clinical and pathological characteristics of DLC might be important factors distinguishing themselves from CHB and LC. That was consistent with some clinical observation. On study of HBV-related liver fibrosis progression, we compared PBMC DNA methylome changes of CHB with LC. IPA pathway data showed that fibrosis progression caused epigenetic reprogramming of the immune and inflammatory response, which providing potential targets for the treatment of liver fibrosis.

## Materials and methods

### Study sample

In this study, 48 HBV-related disease patients who were admitted into Beijing You’An Hospital, Capital Medical from August 23, 2013 to March 1, 2017 were recruited. Forty-eight patients included CHB consisting of 10 patients with fibrosis S1, 9 S2, and 4 S3; 14 LC consisting of 4 S3-4 and 10 S4; and 10 DLC. All patients and their family signed informed consents, and ethical approval was granted from the respective ethics committees at the Beijing You’An Hospital, Capital Medical University (EC-B-031-A01-V9.1-2017-26).

### Illumina HumanMethylation450 BeadChip analysis

After extraction, PBMC DNA was treated by bisulfite converted (ZYMO Research, Irvine, USA), and prepared for Illumina HumanMethylation 450 K BeadChip (Illumina, Inc., San Diego, USA) analysis by CapitalBio Technology according the manufactures’ guide. In brief, bisulfite-converted genomic DNA was whole-genome isothermally amplified at 37 °C for 23 h, and enzymatically fragmented, precipitated, denatured, and hybridized on the BeadChips for 18 h at 48 °C. Then BeadChips were washed, extended with biotin modified ddCTPs, ddGTP or DNP modified ddATP, ddTTP prior to scanning with Illumina iScan system. Preprocessing of the methylation data including raw.idat file loading, filtering out probes located in chromosome X and Y, quality check, normalization, and batch effect correction was performed with Bioconductor package Chip Analysis Methylation Pipeline (ChAMP) implemented in R [38]. Raw data were filtered the probes with detection *P* value > 0.01, with a beadcount < 3 in at least 5% of samples, containing single nucleotide polymorphisms (SNPs) aligning to multiple locations, locating on X,Y chromosome as well as was NOCpGs.

### STEM analysis

We investigated the continuous change of CpG methylation over 5 disease development stages by applying short time-series expression miner (STEM) analysis [39]. For our analysis, mean CpG sites beta value of each group was used for STEM analysis. Firstly, the clustering algorithm was set as STEM Clustering Method to create 50 model profiles, all these model profiles were defined independently of the data from the experiment. Then, STEM used the hypergeometric distribution to compute the significance of overlap between the data from the experiment and model profiles. Filtering parameters were adjusted for CpG methylation data, as difference was set to from 0 to 1 and minimum absolute expression change was set to 0.

### PCR and pyromarksequencing

Bisulfite-converted DNAs were amplified by PCR primers designed with the Pyromark Assay Design software 2.0 (Qiagen, Hilden, Germany) and primers and cycling conditions for pyrosequencing shown in Supplementary table 2. These PCR amplicons were separated and detected by electrophoresis on 2% agarose gel. Briefly, the PCR amplicon for each sample was immobilized by master mix which contained Streptavidin Sepharose High Performance beads (GE, Healthcare) and PyroMark Binding Buffer (Qiagen). The immobilized product was purified by 70% ethanol, PyroMark Denaturation Solution (Qiagen), and PyroMark Wash Buffer (Qiagen) on the PyroMark Q24 Vacuum Workstation (Qiagen), sequentially. Finally, single-stranded DNA was then annealed to a specific sequencing primer (Supplementary table 2) at 80 °C for 2 min, and then cooled to room temperature for at least 5 min. Results were analyzed with the PyroMarkQ24 Software 2.0 (Qiagen).

### Pathway analysis

All CpG sites were linked to genes on basis of only the 450 K BeadChip annotation file. The majority of the significantly differentially methylated CpG sites were located within the gene region, which was categorized into 8 groups: TSS1500 (1500 bp regions upstream of the transcription start site (TSS)), TSS200, 5′untranslated region (UTR), the 1st exon, exon boundaries (ExonBnd), gene body, 3′UTR, and intergenic region. “Promoter” region included TSS1500, TSS200, 5′UTR, and the 1st exon of genes, which was tightly linked to transcriptional progress [28–30, 40, 41]. Selected genes whose “promoter” region containing differentially methylated CpG probes were subjected to QIAGEN’s Ingenuity Pathway Analysis (IPA, QIAGEN Redwood City, www.qiagen.com/ingenuity) to identify relevant immune signaling and functional pathways.

### Software platform and packages

All analyses were carried out using the R 3.5.1 language (http://www.r-project.org/). Correlations between pyromarksequencing and 450 K BeadChip data were tested using Pearson coefficients as implemented in R stats-package. The ChAMP package was for analysis of Illumina BeadChip assay [38] and package Limma [42] was implemented in ChAMP. The R package “glmnet” [15, 16] was for Lasso logistic regression, and “caret” was for cross validation to select the optimal *λ* value of LASSO penalty [43] and confusion matrix analysis of internal testing of LC model. “ROCR” [44] was employed to plot the ROC curve and determine the AUROC.

## Supplementary information

**Additional file 1:.** Supplementary table 1. Distribution of 7888 significantly differentially methylated CpG sites correlated with liver disease progression ( r > 0.8, r < − 0.8; p < 10^−7^)

**Additional file 2:.** Supplementary table 2. Multivariate linear regression of 7888 CpG sites

**Additional file 3:.** Supplementary table 3. Multifactorial ANOVA analysis of 7888 CpG sites

**Additional file 4:.** Supplementary table 4. Primers and cycling conditions for pyrosequencing methylation analysis of candidate CpG sites

**Additional file 5:.** Supplementary table 5. Results of confusion matrix analysis of CpG sites model and LC model

**Additional file 6:.** Supplementary table 6. Multivariate linear regression of 4325 CpG sites

**Additional file 7:.** Supplementary table 7. Multifactorial ANOVA analysis of 4325 CpG sites

**Additional file 8:.** Supplementary table 8. Distribution of significant 4325 CpG sites between LC and CHB

**Additional file 9:.** Supplementary table 9. IPA canonical pathways analysis revealed 113 enriched immune-related pathways between LC and CHB

**Additional file 10:.** Supplementary table 10. Multivariate linear regression of 7888 CpG sites using group and HBV marker

**Additional file 11:.** Supplementary table 11. Multivariate linear regression of 4325 CpG sites using group and HBV marker

## Data Availability

Not applicable.

## Abbreviations

HBV: Hepatitis B virus
PBMC: Peripheral blood mononuclear cell
CHB: Chronic hepatitis B
LC: Compensated liver cirrhosis
DLC: Decompensated liver cirrhosis
STEM: Short time-series expression miner
LASSO: Least absolute shrinkage and selection operator
CV: Cross-validation
AUROC: Area under receiver operating characteristic curve
IPA: Ingenuity Pathway Analysis

## References

[1] Marcellin P (2009). Hepatitis B and hepatitis C in 2009. Liver Int.

[2] Hyun JJ, Seo YS, Yoon E, Kim TH, Kim DJ, Kang HS, Jung ES, Kim JH, An H, Kim JH (2012). Comparison of the efficacies of lamivudine versus entecavir in patients with hepatitis B virus-related decompensated cirrhosis. Liver Int.

[3] Soresi M, Giannitrapani L, Cervello M, Licata A, Montalto G (2014). Non invasive tools for the diagnosis of liver cirrhosis. World J Gastroenterol.

[4] Chinese Society of Infectious Diseases and Chinese Society of Hepatology, Chinese Medical Association. Guidelines for the prevention and treatment of chronic hepatitis B (version 2019). J Clin Hepatol. 2019;35(12):2648–69.

[5] Terrault NA, Lok ASF, McMahon BJ, Chang K-M, Hwang JP, Jonas MM, Brown RS, Bzowej NH, Wong JB (2018). Update on prevention, diagnosis, and treatment of chronic hepatitis B: AASLD 2018 hepatitis B guidance. Hepatology (Baltimore, Md).

[6] Bravo AA, Sheth SG, Chopra S (2001). Liver biopsy. N Engl J Med.

[7] Marie-Christine R, Sophie M, Florence D, Anne C, Pierre B, Jean-Paul S-A, Paul C (2005). Sources of variability in histological scoring of chronic viral hepatitis. Hepatology.

[8] Jaenisch R, Bird A (2003). Epigenetic regulation of gene expression: how the genome integrates intrinsic and environmental signals. Nat Genet.

[9] Zeybel M, Vatansever S, Hardy T, Sarı AA, Cakalağaoğlu F, Avcı A, Zeybel GL, Karahüseyinoğlu S, Bashton M, Mathers JC (2016). DNA methylation profiling identifies novel markers of progression in hepatitis B-related chronic liver disease. Clin Epigenet.

[10] Zhang Y, Petropoulos S, Liu J, Cheishvili D, Zhou R, Dymov S, Li K, Li N, Szyf M (2018). The signature of liver cancer in immune cells DNA methylation. Clin Epigenet.

[11] Bibikova M, Barnes B, Tsan C, Ho V, Klotzle B, Le JM, Delano D, Zhang L, Schroth GP, Gunderson KL (2011). High density DNA methylation array with single CpG site resolution. Genomics.

[12] Gerhard GS, Malenica I, Llaci L, Chu X, Petrick AT, Still CD, DiStefano JK (2018). Differentially methylated loci in NAFLD cirrhosis are associated with key signaling pathways. Clin Epigenet.

[13] Houseman EA, Accomando WP, Koestler DC, Christensen BC, Marsit CJ, Nelson HH, Wiencke JK, Kelsey KT (2012). DNA methylation arrays as surrogate measures of cell mixture distribution. BMC Bioinformatics.

[14] Tibshirani R. Regression shrinkage and selection via the LASSO. J Royal Stat Soc Series B (Methodol). 1996;58(1):267–88.

[15] Friedman J, Hastie T, Tibshirani R (2010). Regularization paths for generalized linear models via coordinate descent. J Stat Softw.

[16] Benton MC, Sutherland HG, Macartney-Coxson D, Haupt LM, Lea RA, Griffiths LR (2017). Methylome-wide association study of whole blood DNA in the Norfolk Island isolate identifies robust loci associated with age. Aging (Albany NY).

[17] Stone GW, Maehara A, Lansky AJ, de Bruyne B, Cristea E, Mintz GS, Mehran R, McPherson J, Farhat N, Marso SP (2011). A prospective natural-history study of coronary atherosclerosis. N Engl J Med.

[18] Li T-Y, Yang Y, Zhou G, Tu Z-K (2019). Immune suppression in chronic hepatitis B infection associated liver disease: a review. World J Gastroenterol.

[19] Uslusoy HS, Nak SG, Gülten M (2011). Noninvasive predictors for liver fibrosis in patients with nonalcoholic steatohepatitis. World J Hepatol.

[20] Boyacioglu S, Gur G, Yilmaz U, Korkmaz M, Demirhan B, Bilezikci B, Ozdemir N (2004). Investigation of possible clinical and laboratory predictors of liver fibrosis in hemodialysis patients infected with hepatitis C virus. Transplant Proc.

[21] Jiao J, Ooka K, Fey H, Fiel MI, Rahmman AH, Kojima K, Hoshida Y, Chen X, de Paula T, Vetter D (2016). Interleukin-15 receptor α on hepatic stellate cells regulates hepatic fibrogenesis in mice. J Hepatology.

[22] Zeybel M, Mann DA, Mann J (2013). Epigenetic modifications as new targets for liver disease therapies. J Hepatol.

[23] Hu G, Wei B, Wang L, Wang L, Kong D, Jin Y, Sun Z (2015). Analysis of gene expression profiles associated with glioma progression. Mol Med Rep.

[24] Ramos PS, Zimmerman KD, Haddad S, Langefeld CD, Medsger TA, Feghali-Bostwick CA (2019). Integrative analysis of DNA methylation in discordant twins unveils distinct architectures of systemic sclerosis subsets. Clin Epigenet.

[25] Luo C, Hajkova P, Ecker JR (2018). Dynamic DNA methylation: in the right place at the right time. Science.

[26] Mishra NK, Guda C (2017). Genome-wide DNA methylation analysis reveals molecular subtypes of pancreatic cancer. Oncotarget.

[27] Gutierrez-Arcelus M, Lappalainen T, Montgomery SB, Buil A, Ongen H, Yurovsky A, Bryois J, Giger T, Romano L, Planchon A (2013). Passive and active DNA methylation and the interplay with genetic variation in gene regulation. Elife.

[28] Brenet F, Moh M, Funk P, Feierstein E, Viale AJ, Socci ND, Scandura JM (2011). DNA methylation of the first exon is tightly linked to transcriptional silencing. Plos One.

[29] Bieberstein Nicole I, Carrillo Oesterreich F, Straube K, Neugebauer Karla M (2012). First exon length controls active chromatin signatures and transcription. Cell Reports.

[30] Brönneke S, Brückner B, Peters N, Bosch TCG, Stäb F, Wenck H, Hagemann S, Winnefeld M (2012). DNA methylation regulates lineage-specifying genes in primary lymphatic and blood endothelial cells. Angiogenesis.

[31] Chang CM, Lo CH, Shih YM, Chen Y, Wu PY, Tsuneyama K, Roffler SR, Tao MH (2010). Treatment of hepatocellular carcinoma with adeno-associated virus encoding interleukin-15 superagonist. Hum Gene Ther.

[32] Su T-H, Kao J-H, Liu C-J (2014). Molecular mechanism and treatment of viral hepatitis-related liver fibrosis. IntJ Mol Sci.

[33] Tan Z, Qian X, Jiang R, Liu Q, Wang Y, Chen C, Wang X, Ryffel B, Sun B (2013). IL-17A plays a critical role in the pathogenesis of liver fibrosis through hepatic stellate cell activation. J Immunol.

[34] Lemmers A, Moreno C, Gustot T, Marechal R, Degre D, Demetter P, de Nadai P, Geerts A, Quertinmont E, Vercruysse V (2009). The interleukin-17 pathway is involved in human alcoholic liver disease. Hepatology.

[35] Zimmermann HW, Seidler S, Gassler N, Nattermann J, Luedde T, Trautwein C, Tacke F (2011). Interleukin-8 is activated in patients with chronic liver diseases and associated with hepatic macrophage accumulation in human liver fibrosis. Plos One.

[36] Svegliati-Baroni G, Saccomanno S, van Goor H, Jansen P, Benedetti A, Moshage H (2001). Involvement of reactive oxygen species and nitric oxide radicals in activation and proliferation of rat hepatic stellate cells. Liver.

[37] Wald O, Pappo O, Safadi R, Dagan-Berger M, Beider K, Wald H, Franitza S, Weiss I, Avniel S, Boaz P (2004). Involvement of the CXCL12/CXCR4 pathway in the advanced liver disease that is associated with hepatitis C virus or hepatitis B virus. Eur J Immunol.

[38] Morris TJ, Butcher LM, Feber A, Teschendorff AE, Chakravarthy AR, Wojdacz TK, Beck S (2014). ChAMP: 450 k chip analysis methylation pipeline. Bioinformatics.

[39] Ernst J, Bar-Joseph Z (2006). STEM: a tool for the analysis of short time series gene expression data. BMC Bioinformatics.

[40] Farkas SA, Milutin-Gašperov N, Grce M, Nilsson TK (2013). Genome-wide DNA methylation assay reveals novel candidate biomarker genes in cervical cancer. Epigenetics.

[41] Wei J, Li G, Zhang J, Zhou Y, Dang S, Chen H, Wu Q, Liu M (2016). Integrated analysis of genome-wide DNA methylation and gene expression profiles identifies potential novel biomarkers of rectal cancer. Oncotarget.

[42] Smyth GK, Michaud J, Scott HS (2005). Use of within-array replicate spots for assessing differential expression in microarray experiments. Bioinformatics.

[43] Xu H, Zhao X, Shi Y, Li X, Qian Y, Zou J, Yi H, Huang H, Guan J, Yin S (2019). Development and validation of a simple-to-use clinical nomogram for predicting obstructive sleep apnea. BMC Pulmonary Medicine.

[44] Sing T, Sander O, Beerenwinkel N, Lengauer T (2005). ROCR: visualizing classifier performance in R. Bioinformatics.

