# Supplementary material for: The signature of HBV-related liver disease in peripheral blood mononuclear cell DNA methylation
Source: Clin Epigenetics. 2020 Jun 8;12:81. doi: 10.1186/s13148-020-00847-z (PMC7278209; doi:10.1186/s13148-020-00847-z)
Supplement: Supplementary file 1 — Additional file 1:. Supplementary table 1. Distribution of 7888 significantly differentially methylated CpG sites correlated with liver disease progression ( r > 0.8, r < − 0.8; p < 10−7) [file 13148_2020_847_MOESM1_ESM.docx]

| Supplementary table 1. Distribution of 7888 significantly differentially methylated CpG sites correlated with liver disease progression ( r > 0.8, r < − 0.8; p < 10^−7^) | | | | | | | | | | | |
| --- | --- | --- | --- | --- | --- | --- | --- | --- | --- | --- | --- |
|  | TSS1500 | TSS200 | 5'UT R | 1stExon | Body | 3'UTR | sum of gene region | sum of “promotor” region | "promotor "/gene(%) | intergenic region | sum of significant CGs |
| significant CGs | 1145 | 973 | 739 | 600 | 2228 | 207 | 5892 | 3457 | 58.67% | 1996 | 7888 |
| Hypermethylation | 293  (25.59%) | 90  (9.25%) | 180  (24.36%) | 59  (9.83%) | 1211  (57.35%) | 152  (73.43%) | 1985  (33.69%) | 622  (17.99%) | 31.33% | 860  (43.09%) | 2845  (36.06%) |
| Hypomethylation | 852 | 883 | 559 | 541 | 1017 | 55 | 3907 | 2835 | 72.56% | 1136 | 5043 |
