# Supplementary material for: The signature of HBV-related liver disease in peripheral blood mononuclear cell DNA methylation
Source: Clin Epigenetics. 2020 Jun 8;12:81. doi: 10.1186/s13148-020-00847-z (PMC7278209; doi:10.1186/s13148-020-00847-z)
Supplement: Supplementary file 4 — Additional file 4:. Supplementary table 4. Primers and cycling conditions for pyrosequencing methylation analysis of candidate CpG sites [file 13148_2020_847_MOESM4_ESM.docx]

| Supplementary table 4.  Primers and cycling conditions for pyrosequencing methylation analysis of candidate CpG sites | | | | |
| --- | --- | --- | --- | --- |
| CpG sites | Forward primer | Reverse primer (5’ biotinylated) | Sequencing primer | cycling conditions |
| cg17149911  (*AAK1*) | GATTTAAAAATTGAGGTTTAGATAAGTGT | ATAATAAAATAAAATTTTATTTT | ATTTAAAAAAATTATATAAAAATTTAA | 95°C 5min  95°C 20sec  45X  58°C 30sec  72°C 30sec  72°C 5min |
| cg05650055 (*MYEOV* ) | GTTTTTTTTGGGGATAGGGATT | CACCCTTATCTCCCTTATTTCTC | GTTGGATATTTTGTGGAGGG |  |
| cg20332088(no gene can be mapped) | AGGTGTYGATATTTTTTTGATTTTAG | CTATTCCCAATACCACACAAAATAC | ATTTATATATTTGTGTAAATATATTTAT |  |
