# Supplementary material for: The signature of HBV-related liver disease in peripheral blood mononuclear cell DNA methylation
Source: Clin Epigenetics. 2020 Jun 8;12:81. doi: 10.1186/s13148-020-00847-z (PMC7278209; doi:10.1186/s13148-020-00847-z)
Supplement: Supplementary file 5 — Additional file 5:. Supplementary table 5. Results of confusion matrix analysis of CpG sites model and LC model [file 13148_2020_847_MOESM5_ESM.docx]

Supplementary table 5.

Results of confusion matrix analysis of CpG sites model and LC model

Confusion matrix and statistics of CpG sites (cg17149911+ cg05650055) model

Reference

Prediction LC non-LC

LC 11 4

non-LC 3 30

Accuracy: 0.8542

95% CI: (0.7224, 0.9393)

No Information Rate: 0.7083

P-Value [Acc > NIR]: 0.01521

Kappa: 0.6543

Sensitivity: 0.7857

Specificity: 0.8824

Pos Pred Value: 0.7333

Neg Pred Valu: 0.9091

Prevalence: 0.2917

Detection Rate: 0.2292

Detection Prevalence: 0.3125

Balanced Accuracy: 0.8340

'Positive' Class: LC

Confusion matrix and statistics of and LC model.

Reference

Prediction LC non-LC

LC 12 2

non-LC 2 32

Accuracy: 0.9167

95% CI: (0.8002, 0.9768)

No Information Rate: 0.7083

P-Value [Acc > NIR]: 0.0004543

Kappa: 0.7983

Sensitivity: 0.8571

Specificity: 0.9412

Pos Pred Valu : 0.8571

Neg Pred Value: 0.9412

Prevalence: 0.2917

Detection Rate: 0.2500

Detection Prevalence: 0.2917

Balanced Accuracy: 0.8992

'Positive' Class: LC
