# Supplementary material for: The signature of HBV-related liver disease in peripheral blood mononuclear cell DNA methylation
Source: Clin Epigenetics. 2020 Jun 8;12:81. doi: 10.1186/s13148-020-00847-z (PMC7278209; doi:10.1186/s13148-020-00847-z)
Supplement: Supplementary file 8 — Additional file 8:. Supplementary table 8. Distribution of significant 4325 CpG sites between LC and CHB [file 13148_2020_847_MOESM8_ESM.docx]

| Supplementary table 4. Distribution of significant 4325 CpG sites between LC and CHB. | | | | | | | | | | | |
| --- | --- | --- | --- | --- | --- | --- | --- | --- | --- | --- | --- |
|  | TSS  1500 | TSS  200 | 5'  UTR | 1^st^  Exon | Body | 3'  UTR | sum of gene region | sum of “promotor” region | “promotor” /gene(%) | intergenic region | sum of significant CGs |
| significant CGs | 597 | 492 | 388 | 212 | 1367 | 147 | 3203 | 1689 | 52.73% | 1122 | 4325 |
| hypermethylation | 275  (46.06%) | 309 (62.80%) | 206  (53.09%) | 117  (55.19%) | 491  (35.92%) | 48  (32.65%) | 1446  (62.72%) | 907  (53.7%) | 62.72% | 503  (44.83%) | 1949  (45.06%) |
| hypomethylation | 322 | 183 | 182 | 95 | 876 | 99 | 1757 | 782 | 44.51% | 619 | 2376 |
